# Electromagnetic Analysis, Characterization and Discussion of Inductive Transmission Parameters for Titanium Based Housing Materials in Active Medical Implantable Devices

**DOI:** 10.3390/ma11112089

**Published:** 2018-10-25

**Authors:** Waldemar Gruenwald, Mayukh Bhattacharrya, Dirk Jansen, Leonhard Reindl

**Affiliations:** 1 Institute for Applied Research, Offenburg University, 77652 Offenburg, Germany; mayukh.bhattacharyya@hs-offenburg.de (M.B.); dirk.jansen@hs-offenburg.de (D.J.); 2 Department of Microsystems Engineering, Institute of Albert-Ludwigs University, 79098 Freiburg, Germany; reindl@imtek.uni-freiburg.de

**Keywords:** titanium, low frequency, inductive transmission, metallic housing, hermetic sealing, longevity, FEM model, active implantable medical devices

## Abstract

The growing demand for active medical implantable devices requires data and or power links between the implant and the outside world. Every implant has to be encapsulated from the body by a specific housing and one of the most common materials used is titanium or titanium alloy. Titanium thas the necessary properties in terms of mechanical and chemical stability and biocompatibility. However, its electrical conductivity presents a challenge for the electromagnetic transmission of data and power. The proposed paper presents a fast and practical method to determine the necessary transmission parameters for titanium encapsulated implants. Therefore, the basic transformer-transmission-model is used with measured or calculated key values for the inductances. Those are then expanded with correction factors to determine the behavior with the encapsulation. The correction factors are extracted from finite element method simulations. These also enable the analysis of the magnetic field distribution inside of the housing. The simulated transmission properties are very close to the measured values. Additionally, based on lumped elements and magnetic field distribution, the influential parameters are discussed in the paper. The parameter discussion describes how to enhance the transmitted power, data-rate or distance, or to reduce the size of the necessary coils. Finally, an example application demonstrates the usage of the methods.

## 1. Introduction

Active implantable medical devices (AIMDs) have been on the market since the first fully implanted pacemaker in the 1960s. Due to the miniaturization of the electronics and the growing capability of micro-processing units, new markets and applications have been developed. Those include implants for cochlear, retina, muscle-stimulation and body-function-sensing. Additionally to system functionality, the AIMDs require a contactless communication interface. This interface can be realized via optical, ultrasound, capacitive, inductive or radio link [1] (Ch. 1).

To ensure the longevity of the implant and to protect the implant electronics from the corrosive body- fluids, while at the same time the body from the partially poisonous compounds of the implant, a hermetically sealed housing is required [2] (Ch. 1.6). While there are several materials in usage, most of the physicians prefer titanium and titanium alloys. This is due to its mechanical strength, the chemical stability, watertightness and biocompatibility [2,3].

Because of the great importance of inductive- interfaces and titanium housings, this paper aims to describe a simple method to estimate the general behavior of inductive energy and data transmission through a hermetically sealed titanium housing for active implantable medical devices. Similar to [4], the standard transformer model is expanded and used to determine the influence of the housing, which is also separated from the transmission without housing as far as possible. The characteristics of the housing are determined through finite element method (FEM) simulations, which then can be applied to the standard two coil transmission in resonant tuned mode. In addition, the physical boundaries, depending on the physical properties of the housing, are derived from simulations. The results shall enable a pre-prototype determination of the housing possibilities and constraints.

While [5] develops theoretical expressions and [4] uses simulated transfer functions, this paper aims to introduce a new method by determining correction factors. Those can be used directly in a transformer model or given coil setup to estimate the effects of conductive materials for inductive links. Therefore, this paper is structured in the following way. Firstly, the FEM simulation is explained with all necessary parameters with a simple example. Secondly, a real coil model with the main parasitic parameters is presented to determine the parts most impacted by the conductive object. This is shown by FEM simulations and impedance measurements. Thirdly, this model is expanded for transmission parameter estimation and validation for a standard two coil transmission in a resonance tuned mode. Additionally, the magnetic vector field distribution is analyzed to identify the eddy current concentration spots, which may allow optimization of the geometric properties. Furthermore, the identified relevant parameters are discussed to highlight the modification and optimization possibilities. Finally, an application of an implantable infusion pump system demonstrates the implementation of the method. These enable a high data rate transmission system for conductive housings at low frequencies.

## 2. FEM Impedance Parameter Estimation

To calculate magnetic field problems, tools like finite element methods are applied. The Finite Element Method Magnetics (FEMM) [6] used here solves two-dimensional planar and axially symmetric problems for low frequency magnetics, with interfaces to Octave [7], for interactive simulations.

**Figure 1:**
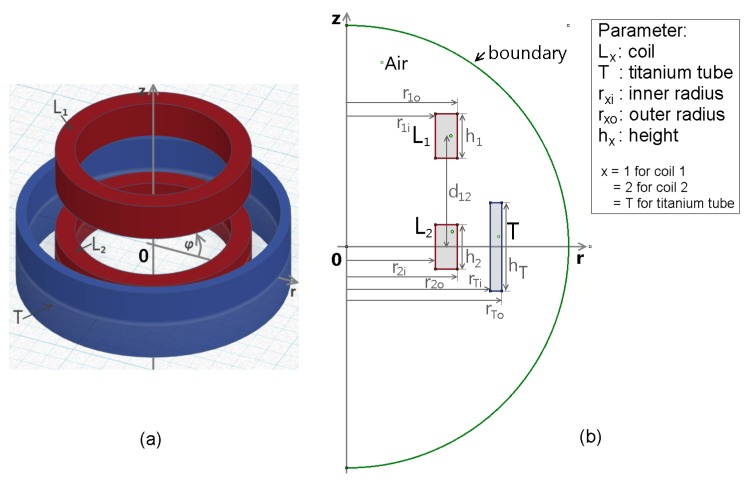
FEM model of an axially symmetric magnetic problem setup, with two coils, $L_{1}$ and $L_{2}$ (red) and a titanium tube *T* around $L_{2}$ (blue). (**a**) shows the three-dimensional model of the setup in a polar coordinate system where *z* is the symmetry axis; (**b**) shows the the setup in FEMM, where the green semicircle is the boundary region and the legend describes the used parameters.

For an axially symmetric problem, as shown in Figure 1a, two coils $L_{1}$ and $L_{2}$ are used (depicted in red), with a titanium tube *T* around $L_{2}$ (depicted in blue). This physical model is translated into FEMM, according to Figure 1b with the necessary geometric dimensions, shown in the parameter box. The problem is enclosed by a spheric boundary (indicated with the green semicircle), where the Robin boundary condition, after [8] (Ch. A.3.2) of the form

(1) $$\frac{\partial \vec{A}}{\partial n}+c_{0}\cdot \vec{A}=0\;\mathrm{with}\;c_{0}=\frac{n}{\mu_{0}\cdot r_{0}}$$

is applied, to approximate an unbounded solution region. In Equation (1), $\partial \vec{A}/\partial n$ is the normal derivative of the magnetic vector potential $\vec{A}$ and $c_{0}$ describes the condition at the boundary, with the harmonic n=1, the magnetic permeability $\mu_{0}=4\pi \cdot 10^{-7}H/m$ and the radius of the boundary circle $r_{0}$. Additional properties for the coils are the number of windings *N*, which are evenly distributed over the cross section of the coil with the wire diameter ${Ø}_{W}$. The conductivity of the coil is $\sigma_{Cu}=58\;\mathrm{MS}/m$ for copper. For the titanium tube, the conductivity is $\sigma_{Ti2}=2.08\bar{3}\;\mathrm{MS}/m$ and the relative permeability is $\mu_{r\;Ti2}=1.000178$.

The simulation is performed while only one coil is excited with the current $I_{i}=1\;A$ and the other coil remains open. The results, taken from the simulation, are basically the impedance parameters. First, the total magnetic flux Ψ=N·Φ, which is the magnetic flux Φ over all windings *N*, can be derived from the magnetic field energy $W_{m}$ [9] (Ch. 9.5), which is

(2) $$W_{m\;i\;j}=\frac{1}{2}\underset{V_{j}}{\;\int \int \int \;}\left({\vec{A}}_{i}\cdot {\vec{J}}_{j}\right)\cdot dV=\Psi_{i\;j}\cdot I_{i}$$

with the volume of the coil *V*, the vector potential $\vec{A}$ and the current density $\vec{J}$.

Case coil 1 is excited with the current $I_{1}$, the magnetic flux in the same coil is $\Psi_{11}=W_{m\;11}/I_{1}$, while the magnetic flux in coil 2 is $\Psi_{12}=W_{m\;12}/I_{1}$ and vice versa. Equation (2) leads to the self inductance $L_{i}$

(3) $$L_{i}=\frac{N_{i}\cdot ℜ\left\{\Phi_{i\;i}\right\}}{I_{i}}=\frac{ℜ\{\Psi_{i\;i}\}}{I_{i}}$$

and the mutual inductance $M_{i\;j}$

(4) $$M_{i\;j}=\frac{N_{j}\cdot \Phi_{i\;j}}{I_{i}}=\frac{\Psi_{i\;j}}{I_{i}}.$$

Next, the induced voltage $U_{i}$, which is the time derivative of Φ, is determined by

(5) $$U_{i}=-N_{i}\cdot ℜ\left\{\frac{d\Phi_{i\;i}}{dt}\right\}=-ℜ\left\{\frac{d\Psi_{i\;i}}{dt}\right\}$$

and used to calculate the coil resistance $R_{i}$

(6) $$R_{i}=\frac{U_{i}}{I_{i}}=-\frac{N_{i}}{I_{i}}\cdot ℜ\left\{\frac{d\Phi_{i\;i}}{dt}\right\}=-\frac{1}{I_{i}}\cdot ℜ\left\{\frac{d\Psi_{i\;i}}{dt}\right\}.$$

Equations (3) and (4) are based on [10] (Ch. 8.10). However, (3) is slightly modified for this case, because only the real part of Ψ is relevant for the inductance, whereas the imaginary part would contribute to the coil resistance. Equation (4) is required to be a complex value, to reproduce the real behavior of the mutual inductance. In addition, Equation (6), which is based on [10] (Ch. 9.1), is modified so that only the real part of Ψ is considered for the resistance calculation.

Finally, the coupling factor $k_{i\;j}$ between two coils can be calculated from Ψ, after [11] (Ch. 3.4.7.3)

(7) $$k_{i\;j}=\frac{\Psi_{i\;j}}{\Psi_{i\;i}}.$$

Based on the FEM simulation without the housing, where the titanium tube is ignored, the basic inductive parameters can be obtained from Equations (3), (4), (6) and (7). Including the titanium tube in the simulation, the primed inductive parameters can be obtained. These provide the necessary values for the further analysis of the impedance and transmission parameters in the following chapters.

## 3. Physical Coil Impedance Model

**Figure 2:**
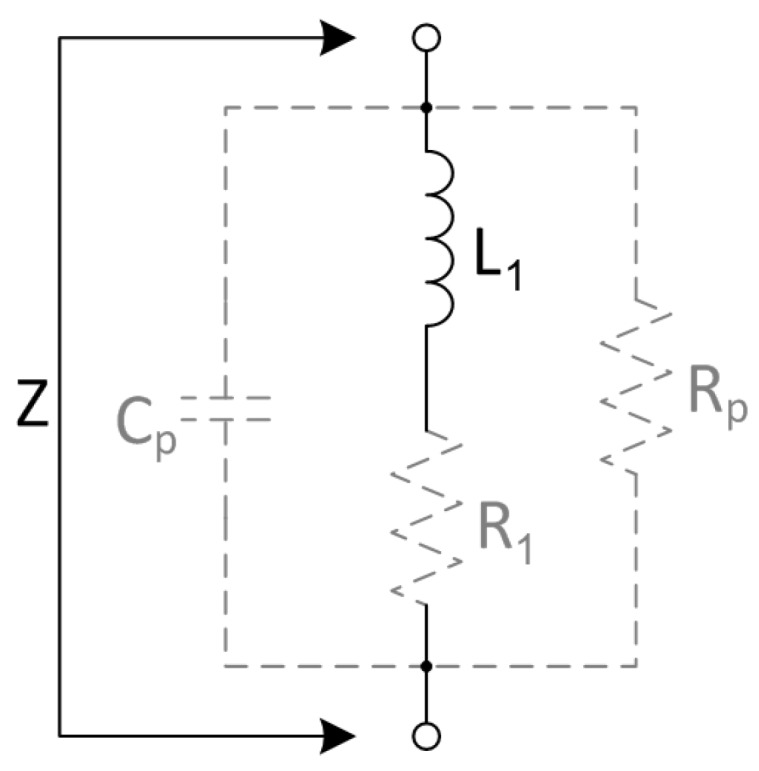
Lumped element model of a simple coil impedance *Z*, consisting of the main inductance $L_{1}$, the equivalent series resistance $R_{1}$, the parallel resistance $R_{p}$ and the parasitic parallel capacitance $C_{p}$.

To describe the frequency dependent properties of a coil, one of the common models is the Resistor Inductor Capacitor (RLC) model [1] (Ch. 2.4). Therein, $L_{1}$ is the main inductance, $R_{1}$ describes the resistive losses and $C_{p}$ describes the parasitic capacitance, which leads to the first resonant frequency. However, this model does not describe the quality factor at the self resonant frequency. Therefore, this model is extended by an additional parallel resistance $R_{p}$. Thus, the electrical behavior from direct current (DC) up to and above the first resonant frequency of the coil can be expressed as a complex impedance *Z*, like shown in Figure 2. This is a slightly modified model of the described large inductor model in [12] (Ch. 5.2). This model is selected because of the practical usability for fast parameter estimation by measurements, which will be explained in Appendix A. However, there is a lot of literature and research on theoretical models, for the estimation of the self-capacitance in [13], the calculation of proximity effects in [14] or the distributive equivalent model of a coil in [15,16].

For the analysis of the impact of the metallic housing or any conductive object in the proximity of the coil, the influence of the impedance of a single coil is analyzed first. Therefore, the housing can be seen as an inductively coupled additional short circuited winding, like in [1,17]. This virtual impedance $Z_{h}=j\omega L_{h}+R_{h}$ consists of $L_{h}$ for the inductance and $R_{h}$ for the resistive losses. Thus, the coupling between the coil and the housing can be treated like a standard transformer with two windings. Those are coupled by the complex coupling factor *k* or the complex mutual inductance *M* (see [18] (Ch. 6)). In the transformer model calculations, it is assumed that only the components $L_{1}$ and $R_{1}$ of the coil are relevant for the coupling. The basic set of equations for the transformer is

(8) $$\left[\begin{array}{c} U_{1}\\ U_{h} \end{array}\right]=\left[\begin{array}{cc} R_{1}+j\omega L_{1} & j\omega M_{1h}\\ j\omega M_{1h} & R_{h}+j\omega L_{h} \end{array}\right]\cdot \left[\begin{array}{c} I_{1}\\ I_{h} \end{array}\right].$$

Because eddy currents run in closed loops, the voltage $U_{h}=0$ allows for eliminating $I_{h}$ and simplify (8), which then can be resolved for the complex impedance $Z^{\prime }$ to:

(9) $$Z^{\prime }\left(j\omega \right)=\frac{U_{1}}{I_{1}}=R_{1}+j\omega L_{1}+\frac{\omega^{2}M_{1h}^{2}}{R_{h}+j\omega L_{h}},$$

where $M_{1h}=k_{1h}\cdot \sqrt{L_{1}\cdot L_{h}}$ is the mutual inductance and $k_{1h}$ is the mutual coupling between the coil and the housing. The impedance can then be separated into the components $Z^{\prime }=R^{\prime }+j\omega L^{\prime }$ with

(10) $$\begin{array}{cc} Z^{\prime }\left(j\omega \right)=\frac{U_{1}}{I_{1}} & =R_{1}+j\omega L_{1}\cdot \left[1-k_{1h}^{2}\cdot \frac{j\omega L_{h}}{R_{h}+j\omega L_{h}}\right]\\ & =R_{1}+j\omega L_{1}\cdot \left[1-k_{1h}^{2}\cdot \chi \right] \end{array}$$

into

(11) $$L_{1}^{\prime }=L_{1}\cdot \left[1-ℜ\left\{k_{1h}^{2}\cdot \chi \right\}\right]$$

and

(12) $$R_{1}^{\prime }=R_{1}+\omega L_{1}\cdot ℑ\left\{k_{1h}^{2}\cdot \chi \right\}$$

with

(13) $$\chi =\frac{j\omega L_{h}}{R_{h}+j\omega L_{h}}.$$

The combined housing parameter χ is a complex value. In combination with the complex coupling factor $k_{1h}$, it contributes to the inductance variation in (11) and the resistance variation in Equation (12). Because of the continuous distribution of the eddy currents and the strong dependency of the physical properties of the housing, it is not possible to simply separate χ and $k_{1h}$. In addition, the housing parameters $L_{h}$ and $R_{h}$ cannot be separated (see also [17]).

However, by extraction of the impedance results from FEM simulations, with and without housing, correction factors can be calculated, which further can be applied to the measured coil values and thus allow an estimation of the influence of the housing. Assuming that the coil parameters $L_{1}$ and $R_{1}$ are constant values, the correction factors can be calculated by

(14) $$\xi_{L}\left(\omega \right)=\frac{L_{1}^{\prime }\left(\omega \right)}{L_{1}}=1-ℜ\left\{k_{1h}^{2}\cdot \chi \right\}$$

and

(15) $$\xi_{R}\left(\omega \right)=\frac{R_{1}^{\prime }\left(\omega \right)}{R_{1}}=1+\frac{\omega L_{1}}{R_{1}}\cdot ℑ\left\{{k^{2}}_{1h}\cdot \chi \right\}.$$

**Figure 3:**
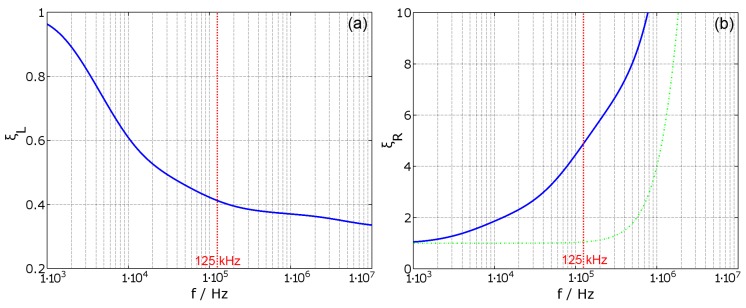
Correction factors $\xi_{L}$ for the main inductance (**a**) and $\xi_{R}$ for the equivalent series resistance (**b**). Additionally, in (**b**), the alternating current (AC) resistance factor $\xi_{Rac}$ without housing is plotted in dashed green, which shows the dominance of the skin- and proximity effect above the frequency $f_{ac}=1\;\mathrm{MHz}$. The parameters, belonging to coil ‘A’ are $N_{A}=200$ and ${Ø}_{W\;A}=0.1\;\mathrm{mm}$ (see also Table A1).

According to Equations (14) and (15), the self inductance $L_{1}$ of the coil is reduced by the conductive object, while the series resistance is increased. These effects are also stated in [5,19]. These calculated factors are displayed in Figure 3, where (a) shows the variation of the inductance and (b) shows the variation of the equivalent series resistance, compared to DC values (shown in bold blue). Additionally, in (b), the frequency dependent variation of a simple air coil is shown as $\xi_{Rac}=R_{1ac}/R_{1dc}$ (represented in dashed green). The parameters of coil ‘A’ are: number of turns $N_{A}=200$, wire diameter ${Ø}_{W\;A}=0.1\;\mathrm{mm}$, coil diameter ${Ø}_{C}=71\;\mathrm{mm}$, coil height $h_{C}=3\;\mathrm{mm}$ and coil width $w_{C}=1\;\mathrm{mm}$. See also Table A1 and Chapter B (The values ${Ø}_{C}$, $h_{C}$ and $w_{C}$ are also used for all other coils in this paper). The skin-effect and the proximity effect inside the coil are dominant above $f_{ac}=1\;\mathrm{MHz}$ and significantly increase the resistance of the coil. Methods for the reduction of the skin- and proximity- effects of the coil are given in [20]. However the focus here is on a frequency of $f_{0}=125\;\mathrm{kHz}$, where these effects are insignificant for the given coil setup. The estimated values of the correction factors are $\xi_{L}\left(f_{0}\right)=0.412$ and $\xi_{R}\left(f_{0}\right)=4.66$. Furthermore, the skin depth for the used material reaches the critical thickness $\delta_{crit}=0.3\;\mathrm{mm}$ of the housing wall at the frequency $f_{\delta \;crit}=1.35\;\mathrm{MHz}$. Above $f_{\delta \;crit},$ a penetration of the housing by magnetic fields is impractical.

Adapted to the coil model from Figure 2, the correction factors can be applied to the values $L_{1}$ and $R_{1}$. Thus, the impedance of the coil inside the housing can be calculated as:

(16) $$Z^{\prime }\left(j\omega \right)=\frac{\left(R_{1}\cdot \xi_{R}+j\omega L_{1}\cdot \xi_{L}\left(\omega \right)\right)}{1+\left(\frac{1}{R_{p}}+j\omega C_{p}\right)\cdot \left(R_{1}\cdot \xi_{R}\left(\omega \right)+j\omega L_{1}\cdot \xi_{L}\left(\omega \right)\right)}.$$

Since one coil is usually tuned to a specific resonance frequency, rather then used in self resonance, Equation (16) is expanded with additional tuning capacitors in series $C_{s}$ and in parallel $C_{p2}$ to

(17) $$Z_{s}^{\prime }\left(j\omega \right)=\frac{1}{j\omega C_{s}}+\frac{\left(R_{1}\cdot \xi_{R}+j\omega L_{1}\cdot \xi_{L}\right)}{1+\left(\frac{1}{R_{p}}+j\omega C_{p}\right)\cdot \left(R_{1}\cdot \xi_{R}+j\omega L_{1}\cdot \xi_{L}\right)}$$

and

(18) $$Z_{p}^{\prime }\left(j\omega \right)=\frac{\left(R_{1}\cdot \xi_{R}+j\omega L_{1}\cdot \xi_{L}\right)}{1+\left(\frac{1}{R_{p}}+j\omega \left(C_{p}+C_{p2}\right)\right)\cdot \left(R_{1}\cdot \xi_{R}+j\omega L_{1}\cdot \xi_{L}\right)}.$$

**Figure 4:**
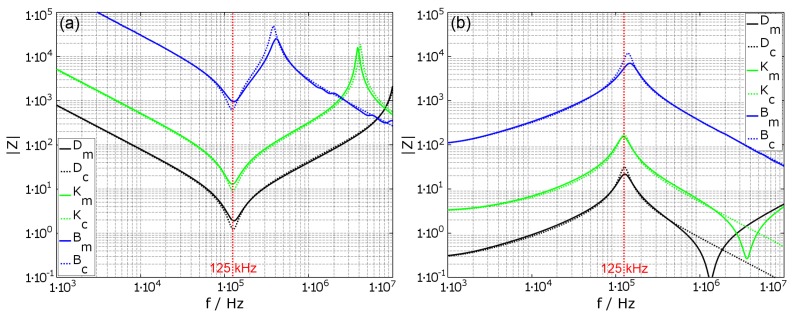
Comparison of the calculated (- -) and measured (–) absolute impedance frequency response for serial (**a**) and parallel (**b**) tuned coils to 125kHz. The different parameters of the coils ‘B’, ‘D’, ‘K’ are $N_{B}=200$, ${Ø}_{W\;B}=0.1\;\mathrm{mm}$, $N_{D}=10$, ${Ø}_{W\;D}=0.4\;\mathrm{mm}$, $N_{K}=25$ and ${Ø}_{W\;K}=0.2\;\mathrm{mm}$ (see also Table A1).

Figure 4 shows the impedance characteristic for coils ‘D’, ‘K’ and ‘B’, which are tuned to a resonance frequency of $f_{0}=125\;\mathrm{kHz}$. In (a), the tuning is in serial mode, according to Equation (17), showing the typical impedance drop at the tuned resonance frequency, but also an impedance peak at higher frequencies. The peak results from the natural resonance of the coil $f_{L1}={\left(2\cdot \pi \cdot \sqrt{L_{1}\cdot C_{p}}\right)}^{-1}$. In (b), the tuning is in parallel mode, according to Equation (18), with the typical impedance peak at the tuned frequency. The impedance drop at higher frequencies and thus the deviation between calculated and measured values is due to additional parasitic components of the system, which are not included in the used coil model. The graphs show that the absolute value of the impedance can be estimated with the parameters. Only at resonance do the calculated values slightly diverge from the measured values. However, the resonance frequency is estimated correctly.

## 4. Transfer Function with Housing

Like the impedance of a single coil, the transfer function between two coils can also be expanded with the simulated housing values. Thus, the transmission of energy and data between two coils in proximity to a conductive material can be estimated; therefore, the standard transformer model for three coils is used, which is based on the system of equations in (19)

(19) $$\left[\begin{array}{c} U_{1}\\ U_{2}\\ U_{h} \end{array}\right]=\left[\begin{array}{ccc} R_{1}+j\omega L_{1} & j\omega M_{12} & j\omega M_{1h}\\ j\omega M_{12} & R_{2}+j\omega L_{2} & j\omega M_{2h}\\ j\omega M_{1h} & j\omega M_{2h} & R_{h}+j\omega L_{h} \end{array}\right]\cdot \left[\begin{array}{c} I_{1}\\ I_{2}\\ I_{h} \end{array}\right].$$

Now, the third winding is used for the housing and is indexed with the character *h*. Again, a symmetric coupling is assumed between the coils and also the housing and so are the mutual inductances. In addition, the voltage of the housing is assumed to be $U_{h}=0$ because of the induced eddy currents. Thus, the current $I_{h}$ can be expressed in terms of $I_{1}$ and $I_{2}$ as

(20) $$I_{h}=\frac{-j\omega M_{1h}}{R_{h}+j\omega L_{h}}\cdot I_{1}+\frac{-j\omega M_{2h}}{R_{h}+j\omega L_{h}}\cdot I_{2}$$

and Equation (19) can be reduced to Equation (21)

(21) $$\left[\begin{array}{c} U_{1}\\ U_{2} \end{array}\right]=\left[\begin{array}{cc} \left(R_{1}+j\omega L_{1}+\frac{\omega^{2}M_{1h}^{2}}{R_{h}+j\omega L_{h}}\right) & \left(j\omega M_{12}+\frac{\omega^{2}M_{1h}M_{2h}}{R_{h}+j\omega L_{h}}\right)\\ \left(j\omega M_{12}+\frac{\omega^{2}M_{1h}M_{2h}}{R_{h}+j\omega L_{h}}\right) & \left(R_{2}+j\omega L_{2}+\frac{\omega^{2}M_{2h}^{2}}{R_{h}+j\omega L_{h}}\right) \end{array}\right]\cdot \left[\begin{array}{c} I_{1}\\ I_{2} \end{array}\right].$$

The previously discussed impedance values from Equations (11) and (12) are still valid for the first coil, but also for the second coil if the index is changed from 1 to 2. In addition, the mutual inductance $M_{12}^{\prime }$ between the first and the second coil can now be expressed as

(22) $$\begin{array}{cc} M_{12}^{\prime } & =M_{12}-\left(\frac{j\omega \cdot M_{1h}\cdot M_{2h}}{R_{h}^{2}+\omega^{2}L_{h}^{2}}\right) \end{array}.$$

$M_{12}^{\prime }$ consists of the mutual inductance between the two coils $M_{12}$, which is affected by the conductive object. By using the coupling coefficient instead of the mutual inductance, Equation (22) can be reformulated in a way, which better shows the influential parts to the coupling:

(23) $$M_{12}^{\prime }=\sqrt{L_{1}\cdot L_{2}}\cdot \left(k_{12}-k_{1h}\cdot k_{2h}\cdot \chi \right).$$

Because $M_{12}^{\prime }$ is a complex value, the real part contributes to the inductive coupling and is reduced by the housing. Likewise, the imaginary part contributes to the resistive losses, which are increased by the housing. Again the correction factor for the housing can be estimated for the mutual coupling. It relies on the coupling coefficients between the housing and the first, respectively, the second coil, divided by the coupling coefficient between the first and the second coil times the complex housing parameter:

(24) $$\xi_{M}=\frac{M_{12}^{\prime }\left(\omega \right)}{M_{12}}=1-\frac{k_{1h}\cdot k_{2h}}{k_{12}}\cdot \chi .$$

**Figure 5:**
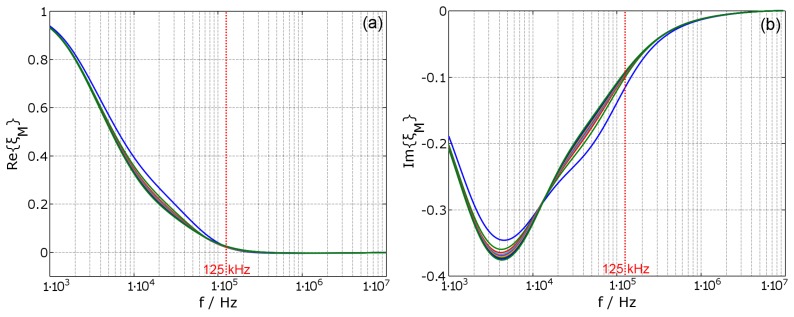
Correction factor for the mutual inductance $\chi_{M}$, split into real part (**a**) and imaginary part (**b**) for coil configuration ‘A’ ($N_{A}=200$ and ${Ø}_{W\;A}=0.1\;\mathrm{mm}$, see also Table A1).

Since the mutual inductance is the main parameter for the transmission, the plot of the correction factor over the frequency is displayed in Figure 5, where (a) shows the real part and (b) shows the imaginary part of the calculated value. The deviation of the values for different distances between the coils increases with decreasing distance of $L_{1}$ to the housing. The value for the frequency of interest is $\xi_{M}\left(f_{0}\right)=0.023-0.115i$.

**Figure 6:**
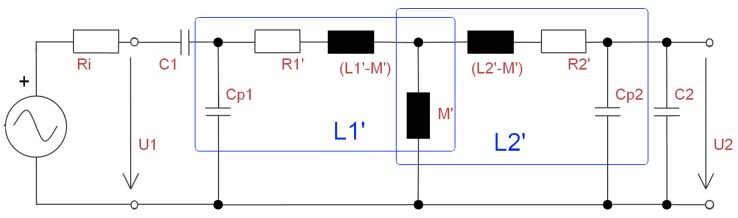
Schematic of typical inductive resonant transmission for the estimation of the transfer function, by using a modified transformer. $L_{1}^{\prime }$ and $L_{2}^{\prime }$ represent the coils, which include the correction factors of the housing.

With these values given, the forward voltage transfer function (FVTF) $v_{f}=U_{2}\;/\;U_{1}$ can be calculated by using the corrected values of $R_{1}^{\prime }$, $L_{1}^{\prime }$ and $M_{12}^{\prime }$. They are composed of the measured or calculated original values multiplied with the frequency dependent values $\xi_{Z}$. In addition, here the dependency is on the geometric arrangement of the housing and the coils. The typical transmission with two coils, where the sending coil $L_{1}$ is tuned in series resonance and the receiving coil is tuned in parallel resonance is depicted in Figure 6.

**Figure 7:**
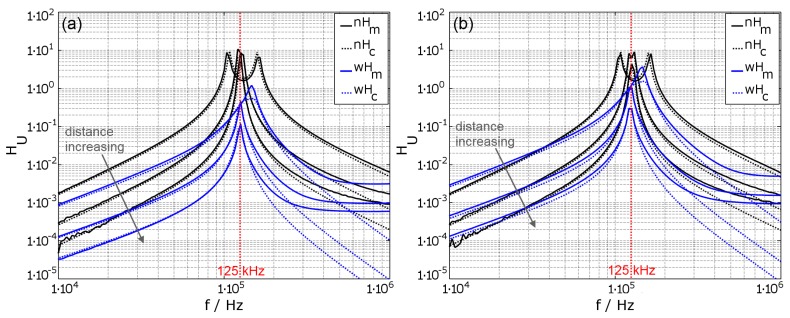
Comparison of the forward voltage transfer function into the implant (**a**) and the reverse voltage transfer function out of the implant (**b**) in a typical inductive transmission without housing (nH) in black and with conductive housing (wH) in blue, – for the measured (m) and - - for the calculated (**c**) results. The used coil parameters for coils ‘A’ and ‘B’ are $N_{B}=N_{A}=200$, ${Ø}_{W\;A}={Ø}_{W\;B}=0.1\;\mathrm{mm}$ (see also Table A1) and the distance between the coils increases from 10mm over 50mm to 90mm.

With the known basic parameters of the coils and the remaining circuit components, the calculated and the measured transfer functions can be compared to each other. Figure 7a shows the FVTF from outside the implant into the implant. Figure 7b shows the backward voltage transfer function (BVTF) $v_{b}=U_{1}\;/\;U_{2}$ from inside the implant to outside the implant. The transfer functions are shown for the case without housing (black graphs, nH) and also for the case with the housing (blue graphs, wH). The distance between the coils is 10mm, 50mm and 90mm. The resonance frequency of the system is tuned to $f_{0}=125\;\mathrm{kHz}$ on both sides, by additional capacitors in series to the sending coil and in parallel to the receiving coil.

For the configuration without housing, the results show that the resonant peak spreads into two peaks after $\omega_{01/02}=\omega_{0}\cdot {\left(1\pm k\right)}^{0.5}$ [4] (Equation (2)), as the coils have a high quality factor. This effect vanishes with increased distance between the coils which also means a reduced coupling factor. The comparison between the measured and the calculated results show good correlation up to about $f\approx 1.7\cdot f_{0}\approx 200\;\mathrm{kHz}$. Above 200kHz, the curves start to deviate because the used model only includes the primary parasitic capacitance and neglects additional parasitic components of the system.

In the configuration with conductive housing, the results are obtained by using the transmission parameters without housing and modifying them with the estimated correction factors from Equations (14), (15) and (24). Since the correction factors only include magnetic field effects up to the first self-resonant frequency, the calculated transfer function starts to deviate from the measurements shortly after the resonant frequency of the system. For most implementations, this is sufficient. For distances of 50mm and 90mm, the calculations are precise; however, for 10mm, the coupling of the outer coil to the housing is not estimated correctly. This results in a smaller value at the resonant peak, but the frequency shift is predicted correctly. In general, the damping of the transfer function increases with the frequency. Furthermore, the resonant frequency increases for small coil distances, due to the additional coupling of the outer coil to the housing. The reduction of the inductance and therefore the resulting resonant frequency shift are compensated by adjustment of additional capacitors.

Since the transfer functions are ratios of received voltage to transmitted voltage in the case with housing, it looks like $v_{f}>v_{b}$, but only because the influence on $U_{2}$ is stronger than the influence on $U_{1}$.

## 5. Eddy Current Distribution in the Housing

In the previous sections, the housing was considered in the form of the influence to the lumped element model of the coil transmission, which is used to tune the transmission and the resonance frequency. However, during the transmission, the magnetic field of the coils induce eddy currents in the housing. Those eddies create an opposing magnetic field, which weakens the original field. Hence, this chapter focuses on the distribution of these magnetic fields. The aim is to find hotspots of the field distribution, which further can be used to redesign the housing or to modify the transmission coils.

**Figure 8:**
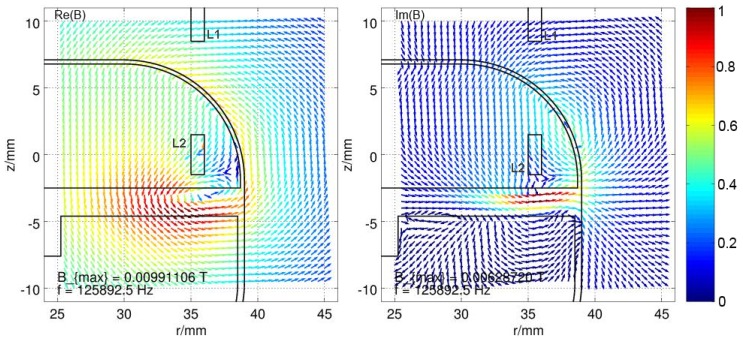
Resulting magnetic field vector distribution, caused by eddy currents in the housing, while exciting $L_{2}$, split into real part (Re(B)) and imaginary part (Im(B)) of the field. The field strength is normalized to the maximum field strength $B_{max}$ and shown in color, according to the colorbar.

**Figure A1:**
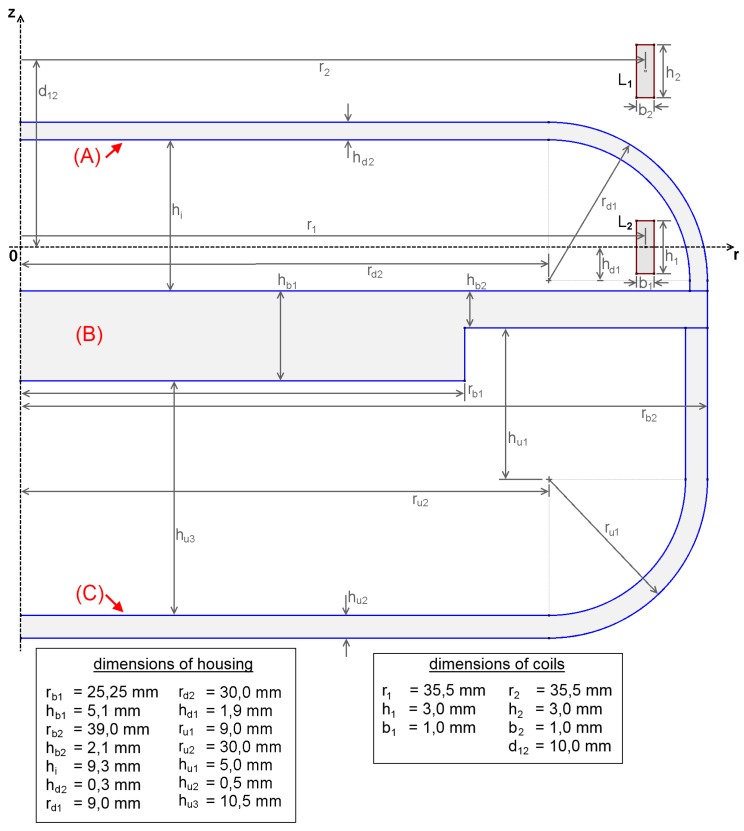
FEM model of the housing and the coils for the estimation of transmission parameters with dimensions, based on a prototype housing of an implantable infusion pump. This model is axially symmetric to the *z*-axis.

Because magnetic fields can be calculated by superposition of different field sources, one can also reverse this effect. The field which results from the eddies in the housing material is simply the subtracted field vectors of the field with housing from the field without, which only consists of two explicit coils. This resulting field distribution for an excited coil $L_{2}$ at a frequency of about 125kHz is displayed in Figure 8. Since the real part is responsible for the influence on the inductance and the imaginary part is responsible for the influence on the resistance of the coils, the real and the imaginary parts of the distributed field are separated. The strength of the field is normalized to the maximum occurring value and represented by a color according to the color bar on the right. The real part of the B field clearly indicates a concentrated magnetic source directly beneath the excited coil. A high concentration of the resulting B field is curling around it with the axis in −φ direction, whereas the current flow in the coil is in the +φ direction. For the imaginary part of the B field, the hotspot appears to be inside the base plate of the housing in the +r direction, directly underneath the excited coil. Both fields oppose the original field and thereby attenuate it, which results in reduced inductance by factor $\xi_{L}$, increased resistance by factor $\xi_{R}$ of the excited coil and also a reduced mutual inductance between the coil by factor $\xi_{M}$. The parameters of the used housing model are described in Appendix B in Figure A1.

**Figure 9:**
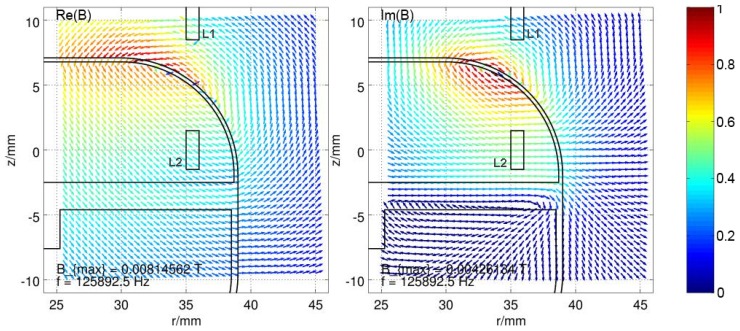
Resulting magnetic field vector distribution, caused by eddy currents in the housing, while exciting $L_{1}$, split into real part (Re(B)) and imaginary part (Im(B)) of the field. The field strength is normalized to the maximum field strength $B_{max}$ and shown by the color, according to the colorbar.

In case the coil $L_{1}$ is excited, the resulting *B* field distribution looks completely different, since now the top cover of the implant housing is the closest conductive part to the coil and since the absolute value of the B field declines with respect to 1/r, where *r* is the distance from the source. The upper cover of the housing has the strongest eddy currents, which reduces the penetration of the *B* field into the housing and therefore also reduces the eddy currents in the base plate (see Figure 9).

## 6. Parameter Discussion

After analyzing the effects of the conductive housing for the transmission, the results can be used to discuss the parameters and further to optimize the inductive link. For eddy currents, the skin effect describes the behavior of the housing on the basis of the skin depth δ, which contains the operational frequency *f*, as well as the magnetic permeability μ and the electric conductivity σ of the used material. It is given by:

(25) $$\delta =\sqrt{\frac{1}{\pi f\mu \sigma }}.$$

According to Equation (25), for example, if the conductivity σ of the housing increases, the same δ is achieved at a lower frequency *f*, which means that the curve progression of the correction factors from Equations (14), (15) and (24) is similarly shifted to lower frequencies with increasing conductivity and vice versa. The same effect takes place, if, instead of the conductivity, the thickness $t_{h}$ increases because the resistance of the housing wall depends on the thickness of the material. These and further parameters are summarized and discussed in Table 1, which is categorized into electrical, coil and housing modifications.

## 7. Realization of a Communication Link for an Implantable Infusion Pump

**Figure 10:**
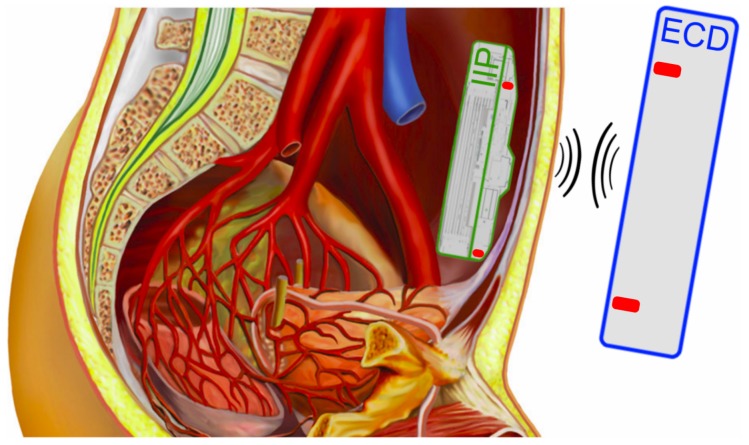
Schematic representation of the inductive link application, with the External Controlling Device (ECD) outside the body (**blue**), the Implantable Infusion Pump (IIP) inside the abdominal cavity (**green**) and the indicated transmission coils (**red**).

In a third party project, the proposed analysis techniques were used for the design of a communication link for an implantable infusion pump. The requirements for the link were to manage the functionality of the implanted infusion pump (IIP) with an external controlling device (ECD). In practice, the IIP is located in the abdominal cavity below the skin. The ECD is placed in the vicinity of the IIP and allows a bidirectional communication with the ECD as a master device, as shown in Figure 10.

**Figure 11:**
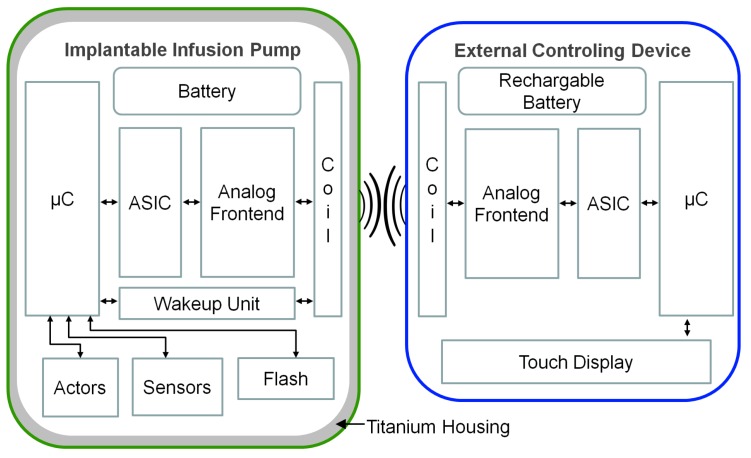
Block diagram of the complete electronics of the implantable infusion pump system, with the IIP on the left side and the ECD on the right side. The main transmission components are the micro-controller (μC), the QPSK-ASIC, the analog frontend circuit and the coil. Additionally, the IIP has sensors, actors, a flash memory and a wake-up unit. The ECD has a touch display, where all commands are handled. Both devices are powered by batteries.

The purpose of the IIP is to deliver a continuous and specific amount of medication to the patient. Therefore, the implant has a drug bellow, surrounded by a tank with pressurized gas, which enables the drug to release. The flow of the medication is controlled by a valve that opens and closes in a timed pattern. Additionally, two pressure sensors allow measuring and adjusting the medication flow and a piezo buzzer can warn the patient in case of any system warning or error. For the data transmission, a DQPSK-ASIC (see [21]) is used in combination with a simple analog circuit and an inductive coil. The carrier frequency of the transmission is $f_{0}=125\;\mathrm{kHz}$. The implant housing is made of grade 2 titanium and hermetically seals the complete interior of the implant, which includes the transmission coil, as shown in the model in Figure A1. This ensures a good receptiveness of the implant inside the patient’s body, since the outer material is biocompatible. Furthermore, the design with the inlaying coil simplifies the development of the implant and also reduces the costs. Although there have been a few attempts for this design, the established data rate and also transmission distance have not been accomplished before. The complete system with all its components is depicted in Figure 11.

The implant electronics are powered by a non rechargeable battery with a capacity of $C_{N}=1600\;\mathrm{mAh}$ and designed for a long lifespan by using low power components for the electric circuits and also keeping the μC in a deep sleep mode for most of the time. Only the real-time clock (RTC) is running permanently, which is responsible for the scheduling of the valve operation. This allows for operating the complete implant electronics at a standby current of $I_{stby}<4\;\mu A$ and hence achieving an operation time of more than eight years. However, the ECD can wake up the implant by sending a specific wake-up packet. The wake-up unit in the IIP is also permanently powered and activates the micro-controller, if it receives this signal. The subsequently exchanged protocol ensures that only authorized devices can access the IIP; otherwise, it goes back to sleep. For transmission, the detuning of the resonance frequency by the metallic housing is compensated according to simulated results.

The shielding effect of the housing also has several advantages. It shields all signals, which are higher than approx. 1 MHz due to the skin effect. This enhances the electromagnetic compatibility of the device. It also dampens the signal strength of lower frequency signals, which only permits a short range wake-up and communication to the IIP. The transmission parameters for the IIP are listed in Table 2.

## 8. Results

The paper presents simple methods to analyze the behavior of a conductive housing for active medical implantable devices with inductive transmission. Section 2 explains the usage of the FEMM tool, in order to simulate the conductive housing influences on the impedance and transmission parameters to the intersection point between magnetic field analysis and lumped element model. Section 3 describes the determination of the essential coil parameters in the form of a modified RLC model with additional parallel resistance for the quality factor. Based on this model, the correction factors $\xi_{L}$ and $\xi_{R}$ for the coil impedance in the vicinity of conductive objects are introduced, which result from the abstracted conductive object χ as an additional short circuited winding. In Section 4, the previous analysis is extended to an actual inductive transmission, based on a standard transformer model, expanded with χ. A new correction factor $\xi_{M}$ is introduced, which enables for determining the transmission parameters, with attenuation and shifting of the resonance frequency. The comparison between measurement of a transmission and simulated results show a good agreement for configurations, where only one coil is affected by the conductive object. In this case, it means that the coil $L_{1}$ is not closer than 10% of the coil diameter to the conductive object. Section 5 focuses on the magnetic field distribution, caused by the housing, which is achieved by the superposition principle. For the analyzed housing, hotspots are in the base plate of the housing directly beneath the implant coil $L_{2}$ for the outwards transmission. For the inwards transmission, by $L_{1},$ the hotspot is in the top cover of the housing. From the results, parameter modifications are derived in Section 6 to discuss the advantages and disadvantages of the transmission of data and power. The discussion is categorized in electrical, coil geometry and housing parameters.

The results are also put in practice in Section 7, where a realization of an implantable infusion pump with external controlling device is described. This system achieves data rates of 10kBits/s for distances of up to 60mm and a longevity of more than eight years and went successfully to market in the year 2014. Until now, these parameters have not been achieved by other products in this sector.

## 9. Conclusions

The proposed analysis techniques enable a fast and easy way to determine the effects of a conductive object in the vicinity of inductively coupled coils for the impedance and transmission parameters. Conductive objects affect the impedance and therefore the transmission between the two coils, which strongly depend on the geometry of the complete transmission system and also on the working frequency. This is due to the frequency dependent eddy current distribution in the object.

The measurements and also the simulations show that the correction factor $\xi_{L}$ for the inductance remains the same for the different coil configurations, as long as the geometry does not change. For the coil, this means that the outer dimensions have to be the same, while the number of turns and also the wire diameter may vary. This specifically allows for generalizing $\xi_{L}$ for certain geometries.

However, this is not the case for $\xi_{R},$ which belongs to the equivalent series resistance (ESR) of the coils due to the strong dependency of the ESR from the current distribution in every winding, which relates to the skin effect, but also due to the inter-winding dependencies in form of proximity effects. Since both effects are frequency dependent, it is not possible to generalize the correction factor $\xi_{R}$ for a certain geometry. Hence, all the effects have to be considered at once and, for every variation in the coil, a new calculation or simulation has to be performed.

The correction factor for the mutual inductance $\xi_{M}$ is a complex value, whereas $\xi_{L}$ and $\xi_{R}$ can be seen as real values, which applies to both the inductance and the resistance of the coils. Therefore, it depends not only on the outer geometry, but also on the number of windings, the width of the coil wire, the space between the wires and also the winding configuration. Hence, the value influences the transmission of the inductance and also of the resistance and has to be simulated specifically for every coil configuration. A generalization is not possible in terms of lumped elements. Instead, it has to be considered on the magnetic field level.

The additional parameter discussion is categorized in three groups: the coil, electrical and housing variations. Therein, principle methods are described to enhance the data rate, distance or the transmitted power and thereby give new perspectives for fast development and implementation.

While the coupling coefficient between two distinct coils purely depends on the geometry of the coils and their arrangement, the coupling between a conductive object and a coil also depends on the distribution of the induced eddy currents in the object, which are strongly dependent on the frequency. Therefore, it is not possible to give general expressions for the coupling coefficient or for the object parameters like *R* and *L*. Nevertheless, the given model is a useful tool for the understanding of the behavior of a conductive object, since it allows for separating the resistive losses in terms of *R* and also the inductive interaction between the coil and the object in terms of *L*.

The FEM model, together with all simulation and calculation scripts are provided in the supplementary files. This FEM model can be expanded with details like the electronics and the printed circuit board (PCB), the medication fluid, gas and the tissue surrounding the housing. However, first measurements show no big deviations from the existing model and also the focus in this paper is on the general behavior of the housing and not specific details, which undergo much stronger variation for each application.

Additionally, this model also allows for modifying the electrical and magnetic properties of the housing material and also for modifying the coil parameters for further investigation and optimization.

## Figures and Tables

**Table 1:** Discussion of advantages and disadvantages of different parameters for titanium encapsulated implantable devices with inductive data and power link.

Type	Parameter	Advantages	Disadvantages
electrical	higher frequency *f*	increased data rate	smaller skin depth δ, therefore higher attenuation and smaller range
	lower frequency *f*	bigger skin depth δ, therefore smaller attenuation and bigger range	decreased data rate
	higher transmission power	increased range	hard to realize inside the implant, because of the limited power source
	higher modulation rank	increased data rate	requires higher bandwidth, or reduces the Signal to Noise Ratio (SNR)
coil	increased geometric coil size	increased range	usually limited by the overall size of the implant and the space inside the housing
	higher quality factor	increased range	reduced bandwidth which leads to lower data rate
	lower quality factor	increased bandwidth which allows higher data rate	smaller range
	using litz wire instead of solid wire for coil windings	reduces proximity and skin effect, which reduces the ohmic losses and increases power transmission	usually more expensive
	intermediate resonant coil outside the housing, under the skin	increases range	more expensive, complicated setup, additional encapsulation necessary
housing	reduced conductivity of the material	increases skin depth, which reduces the attenuation and increases power transmission and range	approval for material usage necessary, eventually reduced mechanical properties
	reduced wall thickness	decrease attenuation by eddy currents, which increases power transmission and range	reduced mechanical strength
	non conductive material	no eddy currents, hence no negative effects of the housing	smaller longevity, biocompatibility and mechanical strength
	reducing hot spots of eddy currents	reduced attenuation, which improves power transmission and range	more complexity in housing and big effort to find the hot spots
	ferrite plate below the coil inside housing	deviation of the magnetic field and reduction of eddy currents, which improves power transmission and range	problematic for Magnetic Resonance Imaging (MRI) scans
	coil outside the housing	improved power transmission and range	lead through and additional encapsulation of coil with non conductive material necessary

**Table 2:** Transmission parameters of the Implantable Infusion Pump (IIP).

Parameter	Description	Value
$U_{in}$	input voltage	1.8V to 3.6V
*f*	operating frequency	12MHz
$f_{0}$	carrier frequency	125kHz
*B*	bandwidth	≈10kHz
*R*	data rate	≈10kBit/s
	modulation	CP-DQPSK
$I_{TX}$	current for sending	<6mA
$I_{stby}$	current in standby	<4μA
*d*	transmission range	10mm to 60mm

## Supplementary Materials

The complete simulation scripts, as well as the FEM models and the measurement data are available online at http://www.mdpi.com/1996-1944/11/11/2089/s1. Refer to README.txt.

## Abbreviations

The following abbreviations are used in this manuscript:

AC	Alternating Current
AIMD	Active Medical Implantable Device
ASIC	Application-Specific Integrated Circuit
BVTF	Backward Voltage Transfer Function
CP	Continuous Phase
DC	Direct Current
DQPSK	Differential Quadrature Phase Shift Keying
ECD	External Controlling Device
ESR	Equivalent Series Resistance
FEM	Finite Element Method
FEMM	Finite Element Method Magnetics [6]
FVTF	Forward Voltage Transfer Function
IIP	Implantable Infusion Pump
MRI	Magnetic Resonance Imaging
PCB	Printed Circuit Board
QPSK	Quadrature Phase Shift Keying
RTC	Real Time Clock
SNR	Signal to Noise Ratio

## Appendix A. Estimation of Coil Parameters by Measurement

By measuring the frequency response of a coil, the parameters for the coil model in Figure 2 can be estimated. The DC resistance of the coil is $R_{1}=ℜ\left\{Z(f_{low})\right\}$, the inductance of the coil is $L_{1}=ℑ\left\{Z(f_{low})\right\}\cdot {\left(2\pi \cdot f_{low}\right)}^{-1}$, where $f_{low}\ll f_{0}$. At the resonance frequency $f_{0},$ the parallel resistance $R_{p}=Z\left(f_{0}\right)$ can be measured because the inductance and the capacitance of the coil cancel out at resonance. The capacitance is then $C_{p}={\left(4\pi^{2}\cdot f_{0}^{2}\cdot L_{1}\right)}^{-1}$. The coils used for the simulation as well as for the experiments are circular air coils with a diameter of ${Ø}_{C}=71\;\mathrm{mm}$ and a height of $h_{C}=3.0\;\mathrm{mm}$ with different wire diameters ${Ø}_{W}$ and number of turns *N*. The parameters, measured via Bode100 analyzer (OMICRON electronics GmbH, Klaus, Austria) [22], are described in Table A1.

**Table A1:** Coil properties.

Name	C	D	H	K	A	B
N	10	10	25	25	200	200
${Ø}_{W}/\mathrm{mm}$	0.4	0.4	0.2	0.2	0.1	0.1
$L_{s}/\mu H$	18.36	18.55	111.4	120.7	6909	6829
$R_{s}/\Omega$	0.2845	0.2818	3.223	3.212	101.1	98.36
$R_{p}/k\Omega$	55.94	55.44	42.44	62.30	312.8	354.8
$C_{p}/pF$	33.18	31.38	51.22	34.88	75.03	67.14
$f_{0}/\mathrm{kHz}$	6448	6598	2107	2453	221.0	235.0

## Appendix B. Description of the FEM Housing Model

The FEM model for the simulation of the housing, which is based on a housing prototype for an implantable infusion pump, is depicted in Figure A1. This model is axially symmetric to the *z*-axis. It consists of the active coils $L_{1}$ outside the housing and $L_{2}$ inside the housing (highlighted in red) and the passive housing (highlighted in blue). The housing itself is made of three parts: (A), (B) and (C). (B) is the base plate, which holds all parts of the implant together. (A) is the top cover, which encloses all electronic components. (C) is the bottom cover, which encloses the drug below and the pressurized gas. The surrounding medium is air. All dimensions are given in the table below the figure. For the simulation, the distance $d_{12}$ between the coils is varied to estimate the coupling behavior. The material, used for the prototype and also the FEM model is grade 2 titanium, with an electrical conductivity $\sigma =2.08\bar{3}\;\mathrm{MS}/m$ (see [23]). The simulation was performed in FEMM 4.2 (see [6]) and controlled and analyzed in Octave (version 4.0.0, written by John W. Eaton and many other) (see [7]).

## Appendix C. Complete Reformulation of the Transformer Equations for Three Windings

(A1) $$\left[\begin{array}{c} U_{1}\\ U_{2} \end{array}\right]=\left[\begin{array}{cc} \left(R_{1}+j\omega L_{1}+\frac{\omega^{2}k_{1h}^{2}L_{1}L_{h}}{R_{h}+j\omega L_{h}}\right) & \left(j\omega k_{12}\sqrt{L_{1}L_{2}}+\frac{\omega^{2}k_{1h}\sqrt{L_{1}L_{h}}k_{2h}\sqrt{L_{2}L_{h}}}{R_{h}+j\omega L_{h}}\right)\\ \left(j\omega k_{12}\sqrt{L_{1}L_{2}}+\frac{\omega^{2}k_{1h}\sqrt{L_{1}L_{h}}k_{2h}\sqrt{L_{2}L_{h}}}{R_{h}+j\omega L_{h}}\right) & \left(R_{2}+j\omega L_{2}+\frac{\omega^{2}k_{2h}^{2}L_{2}L_{h}}{R_{h}+j\omega L_{h}}\right) \end{array}\right]\cdot \left[\begin{array}{c} I_{1}\\ I_{2} \end{array}\right],$$

(A2) $$\left[\begin{array}{c} U_{1}\\ U_{2} \end{array}\right]=\left[\begin{array}{cc} \left(R_{1}+j\omega L_{1}\cdot \left(1-k_{1h}^{2}\cdot \chi \right)\right) & \left(j\omega \sqrt{L_{1}L_{2}}\cdot \left(k_{12}-k_{1h}k_{2h}\cdot \chi \right)\right)\\ \left(j\omega \sqrt{L_{1}L_{2}}\cdot \left(k_{12}-k_{1h}k_{2h}\cdot \chi \right)\right) & \left(R_{2}+j\omega L_{2}\cdot \left(1-k_{2h}^{2}\cdot \chi \right)\right) \end{array}\right]\cdot \left[\begin{array}{c} I_{1}\\ I_{2} \end{array}\right].$$
